# The intellectual structure and substance of the knowledge utilization field: A longitudinal author co-citation analysis, 1945 to 2004

**DOI:** 10.1186/1748-5908-3-49

**Published:** 2008-11-13

**Authors:** Carole A Estabrooks, Linda Derksen, Connie Winther, John N Lavis, Shannon D Scott, Lars Wallin, Joanne Profetto-McGrath

**Affiliations:** 1 Faculty of Nursing, Third Floor Clinical Sciences Building, University of Alberta, Edmonton, Alberta, Canada; 2 Department of Sociology, Vancouver Island University, Nanaimo, British Columbia, Canada; 3 Department of Pediatrics, Faculty of Medicine, University of Alberta, Edmonton, Alberta, Canada; 4 Department of Clinical Epidemiology and Biostatistics and Department of Political Science, McMaster University, Hamilton, Ontario, Canada; 5 Faculty of Nursing, University of Alberta, Edmonton, Alberta, Canada; 6 Department of Neurobiology, Care Sciences and Society, Division of Nursing, Karolinska Institute and Clinical Research Utilization (CRU), Karolinska University Hospital, Stockholm, Sweden; 7 Faculty of Nursing, University of Alberta, Edmonton, Alberta, Canada

## Abstract

**Background:**

It has been argued that science and society are in the midst of a far-reaching renegotiation of the social contract between science and society, with society becoming a far more active partner in the creation of knowledge. On the one hand, new forms of knowledge production are emerging, and on the other, both science and society are experiencing a rapid acceleration in new forms of knowledge utilization. Concomitantly since the Second World War, the science underpinning the knowledge utilization field has had exponential growth. Few in-depth examinations of this field exist, and no comprehensive analyses have used bibliometric methods.

**Methods:**

Using bibliometric analysis, specifically first author co-citation analysis, our group undertook a domain analysis of the knowledge utilization field, tracing its historical development between 1945 and 2004. Our purposes were to map the historical development of knowledge utilization as a field, and to identify the changing intellectual structure of its scientific domains. We analyzed more than 5,000 articles using citation data drawn from the Web of Science^®^. Search terms were combinations of knowledge, research, evidence, guidelines, ideas, science, innovation, technology, information theory and use, utilization, and uptake.

**Results:**

We provide an overview of the intellectual structure and how it changed over six decades. The field does not become large enough to represent with a co-citation map until the mid-1960s. Our findings demonstrate vigorous growth from the mid-1960s through 2004, as well as the emergence of specialized domains reflecting distinct collectives of intellectual activity and thought. Until the mid-1980s, the major domains were focused on innovation diffusion, technology transfer, and knowledge utilization. Beginning slowly in the mid-1980s and then growing rapidly, a fourth scientific domain, evidence-based medicine, emerged. The field is dominated in all decades by one individual, Everett Rogers, and by one paradigm, innovation diffusion.

**Conclusion:**

We conclude that the received view that social science disciplines are in a state where no accepted set of principles or theories guide research (*i.e.*, that they are pre-paradigmatic) could not be supported for this field. Second, we document the emergence of a new domain within the knowledge utilization field, evidence-based medicine. Third, we conclude that Everett Rogers was the dominant figure in the field and, until the emergence of evidence-based medicine, his representation of the general diffusion model was the dominant paradigm in the field.

## Background

The use of knowledge (and science) for the betterment of society is an overarching theme in much of western thought. Knowledge plays such a central role in contemporary societies that they have become known as knowledge societies [1,2]. Many facets of contemporary societies depend increasingly on science and technology [2-4]. Science is not, however, separate from society, and developments in the scientific community are linked to societal changes [5]. How to put knowledge to use is a universal human problem. The problem of putting knowledge to use has been characterized in several ways – for example, as a theory-practice gap [6], as a failure of professionals to adopt evidence-based practices [7], as an inability to bring technological innovations to market [8], and as a lag between discovery and uptake [9,10]. Differences among the various characterizations often occur along disciplinary lines, and along differences in how knowledge is conceptualized, differences in context, and differences in the nature of the producers and users of the knowledge as well as the particular goals each holds within their context. In the health arena, the consequences of not using new knowledge are believed to be dire [11-14], and the agenda of knowledge use has been taken up with vigor – at least among proponents of evidence-based decision-making or evidence informed policy processes.

The field of study in which scholars address these gaps and related issues of importance can be generally labeled knowledge utilization. Many variations in terminology exist, among them innovation diffusion, knowledge translation, research utilization, knowledge mobilization, and technology transfer. These variations commonly signal different groups of scholars and sometimes different disciplines. While these scholars are readily identifiable to those familiar with the field or one of its subfields – despite calls for a discipline of knowledge utilization [15-20], such a discipline has not to date emerged. Although Cottrill, Rogers, and Mills [21] conducted a modified co-citation analysis of 110 authors drawn from the early (1966 to 1972) diffusion of innovation and technology transfer literatures, we could locate no published attempts to map the structure of the scientific community grouped under the rubric of knowledge utilization across disciplines or to map its changes over time.

### Knowledge utilization as a field of study

White, Wellman, and Nazer [22] make the case that objective maps of intellectual structure produced using author co-citation analysis (ACA) have a deep affinity with insiders' perceptions of the structure of their own fields. We held such an insider perception as we began, and that perception is reflected in the following brief overview of the knowledge utilization field and its most obvious subsets (domains). These domains (knowledge utilization, diffusion of innovation, technology transfer, evidence-based medicine or EBM) are, we argue, substantively similar on the basis that they all address the idea of solving social problems with knowledge. They differ along such dimensions as core problems of concern, knowledge used, audiences of relevance, and sometimes modes of transfer.

Rich has argued that the roots of the knowledge utilization field date back to the time of the ancient Greeks [23], although most scholars date it no further back than the earliest studies in innovation diffusion credited to the French sociologist Gabriel Tardé over a century ago [24]. Numerous literatures and traditions (some overlapping) are subsumed within the broad knowledge utilization domain. Some authors have conceptualized knowledge utilization as a broad domain over-arching all others [25,26]. We believe that there has been a strong thread that constitutes knowledge utilization proper whose scholars concern themselves with the relationship of knowledge (often in the form of scientific research) to policy [17,23,27-35]. The most often cited source from this broad overarching knowledge utilization field is Glaser, Abelson, and Garrison's encyclopedic review of the literature on the topic [36]. Backer described the evolution of the knowledge utilization field specifically [37]; Valente and Rogers [38] and Rogers [10] described evolution of the closely related field of innovation diffusion. Beal, Havelock and Rogers offered additional insights into the origins of the field of knowledge utilization, termed by them "knowledge generation, exchange, and utilization" (KGEU) [39]. Havelock argued that the parent discipline of KGEU was sociology, and acknowledged social and organizational psychology as important contributors. Rogers in this same volume clarified the importance of the agricultural extension model and its influence on the thinking of scholars in the field.

### Diffusion of innovations as a field of study

One of the most identifiable domains within a knowledge utilization framework, and until recently the most dominant, is diffusion of innovation. The history of the development of innovation diffusion as a research tradition is well-documented [10,38,40]. Rogers credited the Ryan and Gross classical agricultural study on hybrid corn as creating the template for classical diffusion theory for 40 years [41]. Rogers [10,42] identified nine diffusion research traditions: anthropology, early sociology, rural sociology (dominant until the 1960's), education, public health/medical sociology, communication, marketing, geography, general sociology, and a miscellaneous "other". Valente and Rogers used a Kuhnian framework for their analysis of the rise and fall of the diffusion paradigm among rural sociologists – arguing that the diffusion paradigm faded as a result of a paradigm shift. Although innovation diffusion theory is often described as Rogers' "Theory of Innovation Diffusion", it is more accurate to talk about Rogers' representation of innovation diffusion theory. Crane [40] and Valente and Rogers [38] show that the Ryan and Gross publication formulated the diffusion model. By the mid-1950s, a group of rural sociologists had filled in the major elements. Lionberger's 1960 "Adoption of New Ideas and Practices" [43] contains most of the elements of the diffusion model.

### Technology transfer as a field of study

Technology transfer has a 60-year history of scholarship [44], with interest beginning primarily post World War II, and with periods of heightened interest in the Western world in response to events such as the Cold War, the development of the Space Age, and the emergence of economic competition in the 1970s [45]. In Canada, for example, the role of technology transfer has been spearheaded by the Federal Partners in Technology Transfer, while in the United States a legislative approach has been adopted; these different approaches to technology transfer have subsequently affected each country's progress. For instance, post World War II Canada was slower than its American and British counterparts to establish technology transfer policies [45].

### Evidence-based medicine as a field of study: An emerging emphasis in the health sciences

In 1992 a new group and a new style of knowledge utilization emerged, heralded by the publication of the influential paper "Evidence-Based Medicine: A New Approach to Teaching the Practice of Medicine" [46]. This group of physicians declared a new way of doing medicine – one based on the explicit incorporation of empirical research findings into clinical decision-making processes. Their approach coincided, particularly in the United States, with increasing pressures to manage health care, in large part by reducing variation across both individual and group physician practices. They drew their lineage from the work of epidemiologist Archie Cochrane, who stressed the importance of evaluating medical interventions. Cochrane's work [47] had an important influence on the field of medicine and ultimately resulted in the establishment of the Cochrane Collaboration in 1993. Since the publication of the 1992 EBM manifesto, western society has witnessed a rapid emergence of numerous evidence-based centers, journals and resources.

### Intellectual mapping using citation analyses

Bibliometric analysis (bibliometrics) uses citation data and quantitative analysis to trace published literature and to study the patterns of publication within a field. In analyzing scholarly fields, investigators map structures over time using techniques such as co-citation, co-word, and author co-citation analyses [[48], Chap 1]. In our work, we used ACA in the manner of White and McCain [49].

### What do citations measure?

White and McCain argued that co-citation maps/citation analyses were powerful tools for mapping the intellectual structure of a field over time [50,51]. More recently, they reported longitudinal analyses of the structure and evolution of fields [49,52]. Small proposed that the cited documents are concept symbols [53]. Normative sociologists, among them Zuckerman [54] and Merton [55], viewed citations as markers of intellectual influence and as reward and payment of intellectual debts, respectively. Constructivists Latour [56] and Callon [57] viewed citation as a way of "enrolling allies" to strengthen one's own knowledge claims.

Merton argued that citations denote scholarly influence [58], that they can be used as a measure of scholarly value; they serve the instrumental function of transmitting knowledge, and the symbolic function of rewarding scientists by recognizing their intellectual property rights [59]. In short they are symbolic payment of intellectual debts [60]. Alternatively, constructivists such as Latour [56] have argued that authors use citations to legitimate knowledge claims. By citing another's work, an author strengthens his or her own knowledge claim by tying it to those cited. The social process of making knowledge consists of the successful alignment of initially diverse claims, and if the network is strong enough, the author's knowledge claim becomes an obligatory passage point [57]. Future authors wishing to make claims on the topic must go through this passage point (*i.e.*, the author's work) by citing it. Consistent with Small [60], we argue that both normative and constructivist interpretations of citation patterns are valid.

### Author co-citation analysis

In ACA, cited and co-cited authors are the unit of analysis [51]. As White and Griffith point out, "Co-citation of authors results when someone cites any work by any author along with any work by any other author in a new document of his own" [[61], p. 163]. Spatial maps are produced using one of a number of statistical techniques (*e.g.*, cluster analysis, multi-dimensional scaling, factor analysis). Heavily co-cited authors appear grouped in space, with authors having many links occupying central locations on the maps and authors with weaker links (fewer co-citations) appearing on the periphery of maps [51]. White and McCain argued that ACA simplifies literatures to "writings by use" providing "a more rigorous grouping principle than typical subject indexing, because it depends on repeated statements of connectedness by citers with subject expertise" [[49], p. 329]. Several reports of ACA are available in the literature. White and Griffith covered seven years of the information science literature, finding identifiable author groups, which they call schools [61]. They identified border authors who connect areas of research. White and colleagues recently argued that co-citations reflect intellectual structure more strongly than they reflect social structure [22].

### Invisible colleges

One of the uses to which co-citation analysis is put is the identification of invisible colleges [62,63] – groups of elite, interacting scientists who are geographically dispersed, but who exchange information to monitor progress in their field [40,64,65]. Invisible colleges are generally agreed to represent social networks or significant thought (i.e., cognitive) collectives within a field. The former are commonly studied with sociometric methods, the latter with bibliometric methods. The emergence or strengthening of an invisible college on one hand or the weakening or loss of one altogether on the other, signal important changes scientifically and intellectually – potentially serving to herald significant changes in the ongoing negotiations between science and society of their (sometimes uneasy) social contract. Author co-citation as a method maps intellectual structure, and does not provide direct evidence of social networks in a field.

### Purpose

In the study reported in this paper, we undertook a domain analysis [49,52] using bibliometric methods, specifically ACA to trace historical development of the field of knowledge utilization between 1945 and 2004. Our specific objectives were to map the development over time of knowledge utilization as a scientific field, and to identify the intellectual structure of this scientific community.

## Methods

### Search Strategy

We searched the Web of Science online database covering 1945 to October 2004 with combinations of keywords derived from concepts within the scope of the study (see Additional File 1 for the complete search strategy). Bibliographic information from 14,968 papers was downloaded. The goal of the search was for a balance between recall (exhaustivity) and precision (specificity). Recall is the number of relevant documents retrieved compared to the total relevant documents [66]. Our recall was 88.7%, based upon how many of the possible 200 most cited documents were retrieved in our initial search. Precision is the number of relevant documents retrieved compared to the total documents retrieved [66]. We addressed precision by reviewing all titles and screening for inclusion/exclusion based on pre-determined decision rules. All reviewer pairs had an inter-rater agreement of more than 80%, the first author reviewed the final exclusion decisions; 7,183 titles were excluded. More detailed methods are described in Additional File 2 and further additional information is available in the technical report on request.

### Data Management

We removed 336 duplicates and 3,099 titles that were not "articles" (from the document type field), as articles most often represent new scientific production in a field of study [67,68]. From the initial 14,968 titles, 5,278 articles were retained. Data files were cleaned prior to analysis by correcting for variance in author name, cited author name, cited documents, journal name, and country, and the data were categorized by decade.

### Analysis

Analyses were conducted for each decade starting with 1945. The data were analyzed using Bibexcel freeware, Excel, and Systat 4.0. Descriptive analyses – including most prolific countries, journals, cited authors, and cited documents – were completed by aggregating the data. For co-citation analysis, selection of authors was by frequency of citation. Selection of authors for co-citation analysis can be by a variety of means, such as personal knowledge, review articles, or directories [51,63].

We produced maps for each decade using the twenty-five most cited authors. Twenty-five was chosen as a reasonable number of key authors to produce maps that were interpretable and not visually overwhelming. In one instance (1965 to 1974), 13 authors were chosen, as greater or less than 13 authors produced a map that was not readily interpretable. To create the author co-citation maps, co-citation matrices were first developed from raw citation co-occurrences using Bibexcel. The matrix of co-citation frequencies was entered into Systat 4.0, which uses a multidimensional scaling (MDS) algorithm to find the best-fitting two-dimensional representation of the matrix co-citation entries in the form of a visual map. We assessed the goodness-of-fit of each of the co-citation maps produced using Kruskal's Stress measure [51]. Values for Kruskal's Stress 1 [49] measure were 0.06, 0.16, 0.12, and 0.13 for each of the decades respectively; a stress value less than 0.2 is considered acceptable [51]. We elected to present raw frequency maps because they were more interesting, with variation in the size of the nodes indicating frequency of citation. We reproduced our maps using Salton's cosine normalization [69,70] and found no significant differences or changes to interpretation of the maps. The circles or nodes on the co-citation maps represent frequency of author citations; the lines joining the circles represent author co-citation [51,71]. Thicker lines and closer nodes indicate that the pair are co-cited more frequently, and therefore their work is considered to be conceptually similar [71]. We demonstrated structural change over time by producing a separate map for each decade [72]. The first map is for the decade of 1965 to 1974; prior to that there were insufficient authors to create meaningful maps.

## Results

### Descriptive findings (mapping the field)

#### Domains and countries

The number of distinct domains in which diffusion research occurred increases over time, with the largest increase in the 1995 to 2004 decade. Almost half of the articles (2,363 or 44.7%) identify the United States as their country of origin. The next largest producers are the United Kingdom and Ireland, with 13.1% of the articles (695), and Canada 7.6% (400).

#### Most prolific journals

Table 1 lists the 20 most prolific core journals across all decades, and the total number of knowledge utilization articles published in each between 1945 and 2004. The wide variety in just the top 20 core journals (Table 1) shows a striking degree of inter-disciplinarity. Table 2 represents the five most prolific journals by decade. Between 1955 and 1964, publications in the journal Rural Sociology dominate. This is consistent with accounts that note that until the late 1960s most diffusion research took place in Rural Sociology [10,38]. In the next decade (1965 to 74), most diffusion publications are located in social science journals, and one library science journal. By 1979, the field of knowledge utilization had become sufficiently cohesive to warrant a specialist journal: Knowledge: Creation, Diffusion, Utilization (later called Science Communication). This journal is the core journal in the field for the next two decades. In 1985 to 1994 the Journal of the American Medical Association enters the field of core journals, and in the next decade (1995 to 2004), three of the most prolific journals are health journals.

**Table 1:** Most prolific publishers of knowledge utilization articles (1955 to 2004)

**# of articles**	**Journal Title**
76	Knowledge – Creation Diffusion Utilization*

66	Technovation

60	Journal of Advanced Nursing

59	International Journal of Technology Management

53	British Medical Journal

51	Journal of Evaluation in Clinical Practice

48	Technological Forecasting and Social Change

43	IEEE Transactions on Engineering Management

42	JAMA-Journal of the American Medical Association

41	Research Policy

39	Medical Journal of Australia

32	International Journal of Technology Assessment in Health Care

32	Journal of the American Medical Informatics Association

28	R & D Management

28	Management Science

27	Medical Care

24	Social Science & Medicine

24	Science Communication*

23	Family Practice

23	Journal of General Internal Medicine

*In September 1994 *Knowledge – Creation Diffusion Utilization *became *Science Communication*

**Table 2:** Most prolific journals by decade

Decade	# of articles	Journal title (date of first publication of journal)
1955 to 1964	11	Rural Sociology (1936)

	3	Library Quarterly (1931)

	3	Sociometry (1931)

	2	Social Forces (1922); Personnel Psychology (1948); Review of Economics and Statistics (1917); Human Organization (1941); American Sociological Review (1936); American Documentation (1961); Administrative Science Quarterly (1956)

		

1965 to 1974	8	Nauchno – Tekhnicheskaya Informatsiya Seriya 1 – Organizatsiya I Metodika Informatsionnoi Raboty (1967)

	6	Administrative Science Quarterly (1956)

	5	Human Relations (1947)

	5	Special Libraries (1910)

	5	American Behavioral Scientist (1957)

		

1975 to 1984	35	Knowledge – Creation Diffusion Utilization (1979)

	16	Proceedings of the American Society for Information Science (1964)

	14	R & D Management (1970)

	9	Administrative Science Quarterly (1956)

	9	Rehabilitation Counseling Bulletin (1957)

		

1985 to 1994	41	Knowledge – Creation Diffusion Utilization (1979)

	23	Technological Forecasting and Social Change (1969)

	23	Technovation (1981)

	15	Journal of Scientific & Industrial Research (1942)

	12	JAMA-Journal of the American Medical Association (1883)

		

1995 to 2004	55	International Journal of Technology Management (1986)

	52	Journal of Advanced Nursing (1976)

	51	Journal of Evaluation in Clinical Practice (1995)

	48	British Medical Journal (1840)

	41	Technovation (1981)

#### Most Cited Authors

Table 3 indicates the top-cited authors in each decade in the reference lists of the 5,278 articles in the dataset categorized by decade. Table 4 shows the top-cited document in each decade. The top-cited author in 1945–1954 is H. W. Seinwerth, an industrial relations manager from Chicago in the field of animal husbandry. In 1955 to 64, the top-cited author is Eugene Wilkening, a rural sociologist at the University of Wisconsin, Madison. His technical bulletin on improved farm practices is the top-cited document in this decade, reflecting the prominence of rural sociology in diffusion research at this time. Most citations across all decades (except 1945 to 54) refer to work in the diffusion of innovations field. This field is the parent domain, which arguably provides the conceptual and theoretical core for work in other domains. Everett Rogers is the most-cited author in all decades from 1965 to 2004 (Table 3), and various editions of his book, "Diffusion of Innovations", are the most-cited document from 1964 to 1994 (Table 4). In the last decade, Rogers' book is supplanted as most-cited document by what was to become the index paper for the newly emerging field of EBM [46].

**Table 3:** Most cited authors by decade

**Decades**	**# cites**	**Author name**	**Domain**	**Institution**	**Country**
1945 to 1954	7	Seinwerth, H.W.	Other		USA

1955 to 1964	40	Wilkening, E.A.	Diffusion of innovation, Agriculture, rural sociology	University of Chicago	USA

1965 to 1974	67	Rogers, E.M.	Diffusion of innovation	Ohio State University	USA

1975 to 1984	155	Rogers, E.M.	Diffusion of innovation	Stanford University	USA

1985 to 1994	198	Rogers, E.M.	Diffusion of innovation	University of Southern California	USA

1995 to 2004	627	Rogers, E.M.	Diffusion of innovation	University of New Mexico	USA

**Table 4:** Most cited publications by decade

**Decades**	**# cites**	**Paper**	**Domain**	**Institution**	**Country**
1945 to 1954	-	All cited articles only cited once	-	-	-

1955 to 1964	9	Wilkening, E. A. (1952, May). 'Acceptance of improved farm practices in three coastal plains countries.' *Technical Bulletin 98. North Carolina Agricultural Experiment Station.*	Diffusion of innovation	University of Chicago	USA

1965 to 1974	36	Rogers, E.M. (1962). *Diffusion of Innovations*. First Edition. New York: The Free Press.	Diffusion of innovation	Ohio State University, United States	USA

1975 to 1984	70	Rogers, E.M. & Shoemaker, F.F. (1971). Communication of Innovations: A Cross Cultural Approach.* New York: The Free Press	Diffusion of innovation	Stanford University/University of Denver	USA

1985 to 1994	89	Rogers, E.M. (1983). *Diffusion of Innovations. *Third Edition. New York: The Free Press.	Diffusion of innovation	University of Southern California	USA

1995 to 2004	229	Evidence-based Medicine Working Group (1992). 'Evidence-based medicine. A new approach to teaching the practice of medicine.' *JAMA*, 268(17), 2420–2425.	EBM	McMaster University	Canada

*Note: The second edition of Everett Rogers' 'Diffusion of Innovations' was co-authored with F. Shoemaker and published under the title of 'Communication of Innovations'. Subsequent editions were authored by Rogers only, and published under the name 'Diffusion of Innovations'.

### Longitudinal findings (the intellectual structure)

#### The field over time

**Figure 1:**
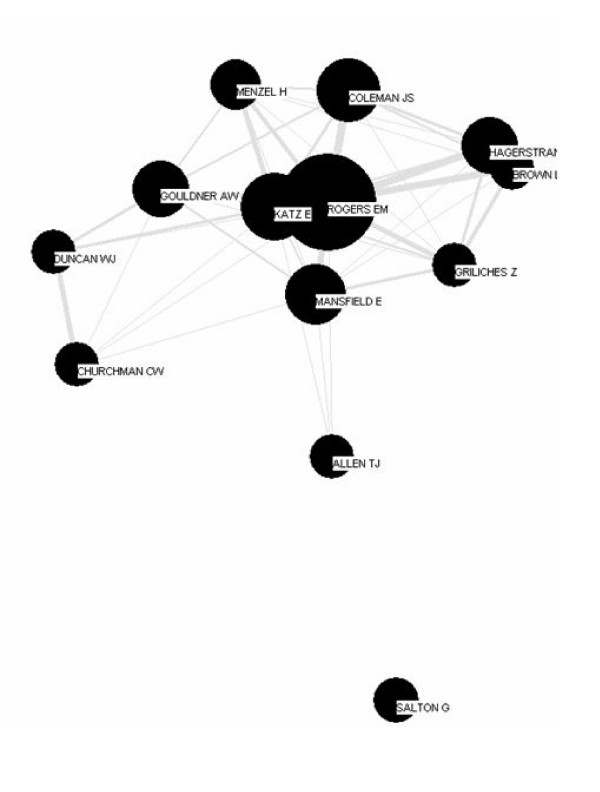
First author co-citation map 1965–1974.

In each decade, new and more robust domains emerged in the knowledge utilization field. A relatively small number of scientists, termed "core sets" by Harry Collins [73,74], played key roles in producing knowledge and resolving scientific controversies in this field. Core sets of scientists are not necessarily in frequent or sustained contact, and we distinguish them from collections of scientists such as invisible colleges who are closely connected. The term helps us to identify a small group of scholars who were actively engaged in the production and certification of knowledge. The core set authors are represented in the maps in Figures 1 through 4, and highlighted in Table 5 by decade. Scholars in the first decade (1965 to 1974) are from diverse disciplines (sociology, economics, geography, management, information science), but are linked by their work in innovation diffusion. Over time they become central figures in distinct subfields which represent their original disciplinary orientation. As noted earlier, prior to 1965 there were too few authors to create meaningful maps.

**Table 5:** Core-set authors by decade by domain

	**1975 to 1984**	**1985 to 1994**	**1995 to 2004**
Knowledge utilization	Caplan	Bozeman	Backer
	Glaser	Backer	Caplan
	Havelock	Caplan	Havelock
	Merton	Dunn	Weiss
	Mitroff	Glaser	
	Rich	Havelock	
	Vandevall	Rich	
	Weiss	Weiss	
	Yin		

Diffusion of innovations	Aiken	Kimberly	Brown
	Brown	March	Coleman
	Coleman	Rogers	Katz
	Downs	Zaltman	Rogers
	Feller		Zaltman
	Hage		
	March		
	Rogers		
	Utterback		
	Zaltman		

Technology transfer	Allen	Allen	Allen
	Federal Insurance	Bass	Mansfield
	Corporation	Jensen	Mahajan
	Mahajan	Mahajan	Nelson
	Mansfield	Mansfield	Rosenberg
	Vernon	Nelson	
		Reinganum	
		Rosenberg	
		Sharif	
		Teece	

Evidence-based medicine		Eddy	Chalmers
		Haynes	Davis
		Lomas	Eddy
			Grimshaw
			Guyatt
			Haynes
			Lomas
			Oxman
			Sackett
			UK Dept Health
			Woolf

Other	Burt		

**Figure 2:**
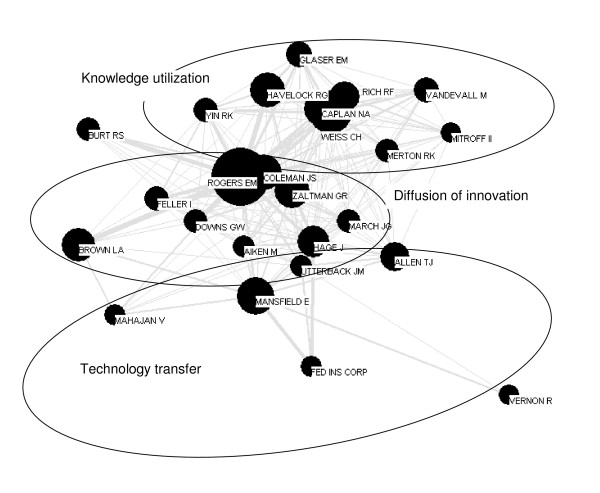
First author co-citation map 1975–1984.

**Figure 3:**
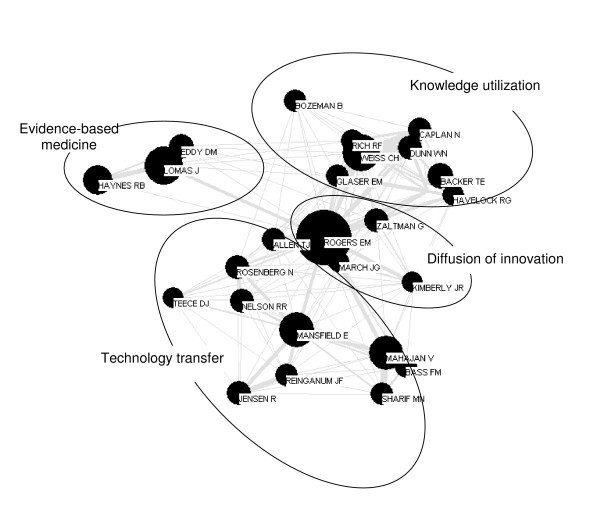
First author co-citation map 1985–1994.

**Figure 4:**
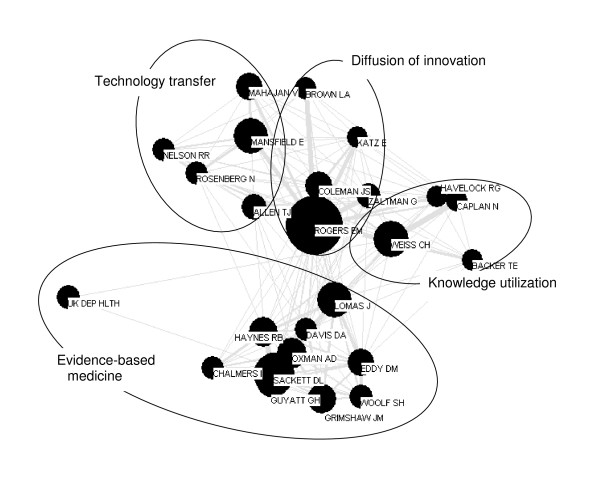
First author co-citation map 1995–2004.

#### 1965 to 1974

Figure 1 shows the core, or parent domain, of diffusion of innovations, characterized by a cohesive [75] group of co-cited authors linked by their common focus on aspects of the diffusion process and the gap between research and practice. The largest and most central node belongs to Everett Rogers, who in this decade published two editions of his groundbreaking work, "Diffusion of Innovations" [76] (the second edition was titled "Communication of Innovations: A Cross Cultural Approach" [77]). This work marks the first analysis of all known diffusion studies [76,77], and the first, and most successful, attempt at articulating a general theory of diffusion. From the outset Rogers' representation of innovation diffusion theory constituted the main paradigm guiding intellectual work in diffusion of innovations.

Sociologist Elihu Katz' work linked disparate fields of diffusion research, such as communication and agricultural innovation [78,79]. Katz' and Rogers' nodes are close to and strongly linked to the nodes of sociologists James S. Coleman and Herbert Menzel, who worked with Katz on the social aspects of the diffusion among doctors of the new antibiotic tetracycline [80,81]. The widely cited study [81] highlighted the importance of interpersonal networks in the diffusion of new medications and was a catalyst for future investigations in this area.

Close to Rogers' node is that of Edwin Mansfield, an economist then writing about the diffusion of innovations in business firms [82-84]. Mansfield's work is also linked to another economist, Zvi Griliches, who examined the economic factors affecting the diffusion of hybrid corn [85]. Thomas J. Allen's work is linked to Rogers through Mansfield. In this period, Allen studied research and development organizations, examining how engineers and scientists communicated and solved problems in organizations [86]. Although all three of these scholars were associated with technology transfer, the content of their work differed [21,87].

To the right of Rogers, and strongly linked to him and to Griliches, are geographers Torsten Hägerstrand and Lawrence Brown, who researched the spatial aspects of diffusion theory [88-90]. Hägerstrand also used Monte Carlo game theory to simulate the diffusion of farm practices [91,92]. To the left of Rogers are sociologist Alvin Gouldner, management theorist W. Jack Duncan and philosopher C. West Churchman. Gouldner [93] studied the differences between "cosmopolitans" and "locals" and the roles that they played in organizations. Duncan studied how to transfer management theory to practice [94], while Churchman studied the gap between managerial decisions and scientific knowledge [95,96]. At the bottom of the map, distant and not linked to the rest of the scholars, is Gerard Salton, an information scientist who examined the link between information dissemination and automatic information systems [97,98].

#### 1975 to 1984

This decade shows a rapid uptake of diffusion scholarship. The parent domain diffusion of innovations grows, and two new domains emerge: knowledge utilization and technology transfer (Figure 2). Rogers' node remains the largest and most central on the map.

#### Knowledge utilization

The conceptual center of this new domain is the work of a new group of scholars – Carol Weiss, Nathan Caplan, and Robert Rich, all of whom investigate the use of social science research in public policy [32,35,99]. They are strongly linked to Rogers and the parent domain of diffusion of innovations. Their nodes are tightly clustered and strongly linked to each other, suggesting a high degree of conceptual similarity.

Moving out from the center are the nodes of Edward Glaser, Ronald Havelock, and Robert Yin. Havelock's early research [19,100,101] examined how knowledge could be used to plan for innovation. Almost 15 years later, Glaser followed on this theme by co-authoring the influential "Putting Knowledge to Use: Facilitating the Diffusion of Knowledge and the Implementation of Planned Change" [36]. Yin's research is conceptually different, focusing on how new practices become routine [102], and the role of networking in knowledge utilization [103]. While Glaser and Havelock were not on the map for the previous decade (1965 to 1974), they were among the most cited authors, appearing on the map when we permitted 50 authors.

On the other side of the central core are Mark van de Vall, Ian Mitroff, and Robert Merton. Van de Vall's work was on the theory and methods used in applying social science research [104,105]. Sociologist of science Ian Mitroff was most cited for his 1974 book "The Subjective Side of Science", where he examines the wide gap between the finished products of scientific work (publications) and the actual processes of forming knowledge [106]. Merton is cited in this decade for the first and revised editions of his book: "Social Theory and Social Structure" [107,108], and for his work on focused interviewing [109]. Merton is fairly strongly linked to fellow sociologist James Coleman, who also wrote on social theory, and received his PhD from Columbia in 1955, where he would have taken courses from Merton.

#### Technology transfer

There is no single conceptual core in this field in this decade, indicated by few links between individuals within the domain, but links back to the domain of diffusion of innovations. This is consistent with the widely differing interests of this core set of authors in the previous decade. Mansfield and Allen have moved in from the parent domain of diffusion of innovation. Mansfield's top citations are to works from the late 1960s and early 1970s that examine the economic aspects of technological change in organizations [110-112]. Allen's most cited work is on research and development laboratories [86,113,114]. Geographer Brown is still strongly linked to that of Rogers in the parent domain, but Brown is also linked to the economist Mansfield through the work of Mahajan. A major contribution of Mahajan and of Peterson and Mansfield was to show how to fit mathematical models to diffusion data.

#### 1985 to 1994

There are three trends in the 1985 to 1994 decade (Figure 3). First is the emergence of EBM as a distinct domain. Second, the domain of diffusion of innovations shrinks in size, although Rogers' node continues to dominate the map (Rogers published another edition of his book in this decade). Third, the knowledge utilization field became more homogeneous and stronger. Two new journals started during the previous decade created arenas in which scholars in knowledge utilization and diffusion could exchange ideas and develop the interdisciplinary application of science knowledge [21]. The emergence of these and other journals and societies are indicators of growing disciplinary cohesion. Authors who remain highly cited in the knowledge utilization domain comprise the current intellectual core set of the field, while authors whose work has not continued to be central to the domain of knowledge utilization exit the map, among them Van de Vall, Mitroff, Merton, and Yin.

#### 1995 to 2004

The map for 1995 to 2004 (Figure 4) shows a continuation of the trends that emerged in the previous decade, especially the growth of EBM. The separate domains show increasing conceptual cohesion internally – citation nodes move closer to each other within the field, and the domains as wholes are more easily distinguishable from the other fields.

At first glance, it appears that the other domains have gotten smaller in this decade. Sociologist James Coleman's early tetracycline study [80] reappears in this decade in the domain of diffusion of innovations. Although White and McCain argue that the reappearance of older work may indicate the revival of a domain [49], we attribute the reappearance of this one work to its relevance to the new EBM project. Coleman is also highly cited in works related to the diffusion of innovations within healthcare [115-117].

In this decade, the most cited article is the index EBM paper [46]; with its spread, the term EBM enters the lexicon. The paper was published in a highly visible and easily accessed medical journal and its author group included 29 members, among them Guyatt, Haynes, Oxman, and Sackett (chair of the group). The authors continued to cite the original paper, toured and gave numerous talks [118-121]. Their work coincided with emerging concerns about rising health care costs and increasing accountability pressures, such as have been described by Nowotny and others [1,122-124].

### Canonical authors and canonical works

White and McCain [49] define a canonical author as someone who appeared on the citation maps in three or more decades. We identify seven canonical authors whose work has enduring importance to the field and who were on at least the last three maps (1975 to 2004). We argue that the most cited works of these authors constitute the canonical literature of the science of knowledge utilization as it entered the twenty-first century. These canonical authors are: Everett Rogers and Gerald Zaltman (innovation diffusion), Carol Weiss and Ronald Havelock (knowledge utilization), and Edwin Mansfield, Thomas Allen, and Vijay Mahajan (technology transfer). Rogers, management scientist Allen and economist Mansfield are the only authors who were top-cited in all decades, excluding 1945 to 1954.

The intellectual structure of the field in all decades is dominated by the work of Everett Rogers. His theory has been the dominant and most consistently used theory since inception of diffusion research [125,126], and is emblematic of the diffusion paradigm until the emergence of EBM in the last decade. We argue that Rogers is a canonical author [49], that his book "Diffusion of Innovations" is a canonical text for all the domains, and that his approach to innovation diffusion represents the dominant paradigm for conducting diffusion research. "Diffusion of Innovations", first published in 1962, went through five editions before his death in 2004 (1971, 1983, 1995, 2003) [10,76,77,127,128]. Each edition of his book was based on an analysis of all retrievable diffusion studies. The presence of such a dominant author stands in contrast to, for example, the information sciences field, characterized by the absence of a strong central author [49]. It also stands in contrast to some of our own canonical authors such as Mansfield, whose early publications are the only work top-cited in all decades.

### Diffusion of innovations

In addition to Everett Rogers' canonical status in this domain, Gerald Zaltman's most cited works were "Innovations and Organizations" [129] and "Strategies for Planned Change" [130]. While Zaltman's work was strongly pro-innovation, it also reflected a belief that innovation should not be accepted unquestionably. He showed the importance of individual users of innovations to the diffusion process, and he proposed that to understand how innovation diffusion truly occurs we need to study demand characteristics, such as users' ability and willingness to seek and process innovations.

### Knowledge utilization

Carol Weiss was most cited for her works "Knowledge Creep and Decision Accretion" [35] and "Using Social Research in Public Policy Making" [131]. She continues to publish [132,133], and has expanded initial views of research use as an instrumental application of research to inform a decision, to include conceptual or enlightened use – when findings from research influence decision makers' attitudes to and perceptions of a social problem [134]. She described several models of utilization: knowledge-driven, problem-solving, interactive, political, tactical, and research as part of the intellectual enterprise of society [34]. An advocate of using research findings to inform public policy, she was among the first to examine the utilization of evaluation findings in improving program processes and program outcomes [135]. Her articulation and extension of the concept "research utilization" was an important contribution to the field of knowledge utilization [34].

Ronald G. Havelock is recognized for his extensive work on knowledge use, change planning, and technology transfer. Author of "Planning for Innovation through the Dissemination and Utilization of Knowledge" [101], his work spanned many fields including education and medicine. Building on Rogers' work [76], Havelock developed a framework which aided in the understanding and improvement of the dissemination and utilization of knowledge in the social sciences. Guided by an extensive analysis of this literature from education and beyond, he proposed an often cited "linkage model" that connects researchers with end users in a two-way exchange of information that mutually enhances problems solving.

### Technology transfer

Edwin Mansfield's early work from the 1960s formed "concept symbols" for the domain [53] and exerted a continuing impact on the field [110-112]. An influential economic analyst of technology, he explored the length of time required for firms to uptake decisions and products used by rival firms, and how this information is spread from one firm to the next. He also studied the proportion of new products and processes that are based on academic research, and the amount of time needed for such research findings to be incorporated into the commercial environment.

Among other contributions, Thomas J. Allen studied the influence of distance on information transfer and developed the Allen curve, which depicts the inverse relationship that exists between distance and the frequency of communication [86,113,136]. Allen also studied the ways in which formal and informal associations within organizations contribute to the diffusion of knowledge. He identified "gatekeepers" as important individuals within organizations who bring new knowledge into their organization both by reading literature and by engaging with others outside of the organization. He found that new ideas are spread most commonly through informal mechanisms such as personal contact.

Vijay Mahajan wrote on developing knowledge in the areas of marketing strategies, product diffusion, and research methodology. He is responsible for adding the temporal element into a model originally designed by Blackman [137] to explain technological substitution. Mahajan has made important contributions in the area of product diffusion and has studied the diffusion process in developing countries, which he argues is an important but understudied area [138,139]. A more detailed version of the longitudinal findings is available in Additional File 3.

## Discussion

We set out to trace the historical development of the field of knowledge utilization by mapping its development over time, and identifying the intellectual structure of this scientific community. The major contribution of this paper is its overview of the field and how the field has changed over time. In this discussion, we contextualize the major findings and expand on four discussion points: development of the field, its specialization, and changing perspectives; our inability to claim that this field (perhaps emerging discipline) is pre-paradigmatic; the emergence of Everett Rogers as a canonical figure in the field; and; the emergence of a new domain, EBM, within the knowledge utilization field.

The maps in Figures 1 through 4, compiled from aggregate author co-citation data, link oeuvres and offer a panorama of the changing intellectual structure of the field, showing the "history of the consensus as to important authors or works" [[50], p. 100]. White and Griffith describe oeuvres as a set of writings by a co-cited author [61]. While an author's node on the map likely represents more than one of their publications, it does not guarantee that all of the author's publications are represented. The only works that will be represented are those that are co-cited with the other authors in the analysis. So, for example, someone might write one article early in their career that is cited for many years, but other works by the same author might not be co-cited.

### Development of the field and its intellectual history

Our first major finding is that new domains within the field now generally referred to in the literature as knowledge utilization have emerged over time; in earlier generations, the term most widely used was innovation diffusion. We argue that although all of the domains we identify are concerned with the use of knowledge in some way, they change and take on distinct specializations and perspectives over time and continue to be strongly linked to innovation diffusion. This finding is in contrast to White and McCain's longitudinal ACA of the information science literature, where they found tremendous inertia, or lack of change over time [49]. They argue that their maps could have looked different at the separate time points if there had been major changes in the field. Our maps do look different in each decade, reflecting continuing change and growth in the intellectual structure of the field. The 1965 map represents authors from a wide variety of academic disciplines whose common object of inquiry is conditions surrounding the use or application of scientific knowledge. In the 1975 to 1984 decade knowledge utilization and technology transfer emerge as distinct areas of study, and in the 1985 to 1994 decade EBM emerges. Over the decades new areas emerged, centered on the work of canonical authors who were already working in the field, before it divided into subfields.

The origins of this broad knowledge utilization field lie in the study of the diffusion of agricultural innovations in rural sociology, credited by Rogers as dating back to Ryan and Gross' hybrid corn study. In a modified co-citation analysis of the diffusion of innovation and technology transfer literature between 1966 and 1972, Cottrill, Rogers, and Mills found that the majority of the members of their diffusion of innovations cluster were from sociology [21]. In Table 4, Wilkening's work on the diffusion of agricultural innovations is the most-cited work in the 1955 to 1964 decade. By the late 1960s, research on the diffusion of innovations in rural sociology had virtually died out, possibly because it solved the particular problem of producing and disseminating means by which high yield crops are produced.

A second important finding is that, over time, the initial core set of authors in diffusion research branched off to become the intellectual center of their new fields. In other words, new fields branch off from the original field of innovation diffusion, and at the core of each of these new fields (with the exception of EBM), we see one of the authors in the first or second decades. Rogers and Mansfield emerge from the first decade as central to innovation diffusion and technology transfer in later decades, respectively. Weiss, Caplan, and Havelock appear in the third decade and remain in each subsequent decade under the knowledge utilization domain. EBM first appears in the 1985 to 1994 decade with three authors, Eddy, Haynes, and Lomas. Lomas is a border author providing the primary connection between EBM and innovation diffusion (and to a lesser extent knowledge utilization) [61].

### Challenging Kuhn's notion of pre-paradigmatic: The diffusion of diffusion

The social sciences have been characterized, most famously by Kuhn, as being "pre-paradigmatic," a state where no accepted set of principles or theories guide research in the area [140]. Although sometimes disputed, Kuhn suggested that the social sciences were characterized by disagreement and lack of consensus, and argued that the natural sciences are characterized by long periods of normal science, where practitioners are guided by a single theoretical model, which aids them in solving puzzles that fit within the paradigm. In 1979 Small and Crane found evidence that the fields of economics, psychology, and sociology were developing in a manner more characteristic of the natural sciences [75]. Kuhn further argued that natural science does not progress in a cumulative fashion, but instead is punctuated by revolutions that radically alter the theoretical rules that inform practice. Here, in the overarching field of knowledge utilization, we find no evidence of the fragmentation and allegiance to multiple-paradigms predicted by Kuhn [140].

Valente and Rogers claim that the pre-paradigm period in innovation diffusion was in the 1930s [38], and that the paradigm was set by Ryan and Gross [141]. Although in 1983 Rogers claims the Ryan and Gross article as the top cited one in diffusion literature [127], our analysis found that that various editions of Rogers' books are by far the highest cited documents in the innovation diffusion literature. Our findings also provide strong evidence that this social science field is more like Kuhn's portrayal of the natural sciences during periods of normal science. There is a paradigm from the beginning – when Rogers publishes the first edition of his book. We believe that Rogers' work had such a significant impact because it was the first, and continued to be the only work, by virtue of its ongoing syntheses, that described (at least his representation of) a general theory of innovation diffusion. Further, Rogers' synthesis of most known empirical studies of diffusion remained useful to scholars.

If Kuhn's claim of the social sciences as pre-paradigmatic is correct, then in knowledge utilization we have an atypical social science field, growing in an atypical way. The maps show growth and specialization over time, not fragmentation as predicted by Kuhn. The knowledge produced about diffusion is taken up and used – it does not languish. People on this map share many assumptions, and their intellectual debt to the work of Everett Rogers shows in the persistent size of his node, and in the strong links to each newly emerged field, including EBM. Zuckerman argued that citations indicate intellectual influence [54]. If so, no single person has been more influential in this field than Rogers; many if not most on this map share an intellectual debt to him. Rogers put forth a representation of a "generalized model of diffusion" [[142], p. 16] in his first book [76] and "set forth common findings to date, arguing for a general diffusion model, and for more standardized ways of adopter categorization" [[142], p. 16].

Although the study of diffusion in rural sociology became exhausted in the 1960s [38], Rogers argued [[142], p. 19] that diffusion research was not dead or dying: "The number of diffusion publications completed per year continues to hold steady. Unlike most models of human behavior that begin to fade after some years of use, the diffusion model continues to attract strong interest from scholars." We also did not find a 'fading' of the diffusion paradigm. Our citation maps show that there is not a shift away from the diffusion paradigm; rather, there is a spread of the paradigm to other fields and areas of specialization. We do see, however, from the titles of the articles of the most-cited authors, a content shift away from the concerns of agriculture.

### The influence of Everett Rogers

Everett Rogers, with the publication of his 1962 book, became an obligatory passage point [57]. His continued publication of updated editions of that book [10,76,77,127,128] ensured that he remained an obligatory passage point making it, we propose, nearly *de rigueur *to cite Rogers when writing in the knowledge utilization field. This may change if EBM continues its explosive growth; we observed that its proponents were less consistent in their citation of Rogers than those in other subfields.

Rogers is generally viewed as one, if not the most influential, social scientists of the last one hundred years. His book is the second most cited in the social sciences [143]. Some reasons for this influence are obvious. He worked in several universities (among them Iowa State, Michigan State, University of Southern California, University of Michigan, University of New Mexico) and had many academic associates, among them some of the original diffusion scholars – Beal, Coleman, Gross, Ryan, and Wilkening. He worked on projects in many countries; he was invited to speak widely and often. He had many graduate students and colleagues and was known for his generosity and gift for bringing people and institutions together. He shared authorship and ideas widely.

Sociologist of science Knorr-Cetina argued that each of the sciences produces knowledge in a different way [144]. For example, in molecular biology, she argued that scientist's biographies affect their epistemology – that through their careers their bodies become finely tuned measurement instruments, learning to perform delicate operations that cannot be taught. In much the same way, there is evidence that Rogers' personality and his way of doing research – his epistemology – was an embodiment of the same networking principles which he studied. His personal biography embodied his theorizing. After Rogers' death, four of his former students recalled their personal associations with him [145]. Thomas Backer recalled that "Ev had a remarkable gift for bringing together people and institutions that otherwise didn't talk to each other much – he was the best example in the world of the kind of natural networker he studied in his research" [[145], p. 291], and well known for his enthusiastic ability to "help connect people he thought should know each other, or who could work with him on a project" [[143], p. 285]. James Dearing, argued that "Perhaps intuitively, Ev understood the social capital advantages of heterophilous relationships" [[145], p. 294]. Thomas Valente wrote that after the first conference on Entertainment Education in 1989, Rogers invited everyone to his house for dinner. It became a "raucous celebration," and he goes on to note that " [e]veryone who was there marks it as the time when entertainment education became a cohesive field of scholarship" [[145], p 297].

Rogers lived a long and productive life. He completed his doctoral work in 1957 at the age of 26, and remained active as a scholar for the next 47 years. He wrote 36 books and more than 350 refereed journal articles [145] including a new edition of his well known "Diffusion of Innovations" about every ten years (1962, 1975, 1983, 1995, 2003). His last published paper appeared posthumously in 2005 [146]. In it, Rogers reflected on his own unique place in the emergence of the diffusion model and on the model's origins in the literature review chapter in his dissertation. Of central importance is that he created a common language with which scholars could talk about diffusion – by emphasizing the term diffusion "rather than the plethora of terms that had been used for this concept" [[142], p. 16]. He concluded that "... it seems there is indeed a general diffusion model" [[142], p. 19].

From a science studies perspective, by studying every known diffusion study regardless of discipline, Rogers unknowingly enrolled thousands of allies to his cause [56]. We do not claim that this was his intent, but by taking something from a wide range of fields (the empirical studies of diffusion), and by giving back something to every field (a general theory of diffusion and a common language), Rogers built a formidable knowledge claim. It is also important to note, however, that citation studies find that authors are cited for their usefulness and for their merit. The story could have unfolded differently if the scholars in the field had not found Rogers' representation of innovation diffusion theory to be of use in solving problems in their own areas. In other words, Rogers is not important just because he studied all known diffusion studies, but because he did this and produced a useful representation of diffusion theory; he got it right, so to speak. His representation of innovation diffusion theory has been shown to be stable and of use to a wide array of scholars in diverse fields. However, getting it right is no guarantee of success. The history of science is replete with people who got it right but who were not credited as such, or people that only time proved were right. Perhaps most famously, in the nineteenth century Pasteur's germ theory won out over Pouchet's theory of spontaneous generation – not because the evidence supported Pasteur, but because in part at least, the members of the French Académie des Sciences were biased in favor of Pasteur. At the time, an "evidence-based" decision would have supported Pouchet's theory [147]. In addition to getting it right, success of the kind that Rogers and the theory of innovation diffusion have earned over the decades is a complex blend of crafting and winning credibility among peers [148], and having one's knowledge claim noticed and taken up by some relevant community [56,74].

### The emergence of evidence-based medicine

In this paper, we have presented a largely descriptive picture of the growth of the field known broadly as innovation diffusion. We have shown that until recently, research in this field has been informed largely by one theoretical paradigm, laid out in the work of innovation diffusion scholar Everett Rogers. We have shown how the field began with a core set of scholars from many disciplines with a common interest in innovation diffusion. We have shown that, over time, this core set of scholars formed the core of new, but related fields. We demonstrated that in the mid-1980s another field emerges (EBM). This field is linked intellectually particularly to Everett Rogers, primarily through the work of Jonathan Lomas, who is strongly linked to Haynes in EBM. The work of Eddy pulls more widely from the field of knowledge utilization, as well as from scholars in the parent domain of innovation diffusion. In the 1990s, we see the field of EBM growing and drawing from the fields of technology transfer, knowledge utilization, and innovation diffusion. Terminology, even in this subfield and within the health disciplines, is complex. For many groups working in the field that we have labeled with the cover term EBM, a broader cover term will be necessary. One sees for example terms such as evidence-based practice, evidence-based nursing, evidence-based decision-making, and evidence-informed decision-making used widely.

We argue that the rapid emergence of this new domain is possible because its adherents practice a form of knowledge production and scientific output that differs significantly from those in related fields. This production of outputs is characterized in particular by an emphasis on systematic reviews of the research literature. Its adherents tend to publish in journals with unusually high impact factors and wide dispersion. Their emphasis is arguably more vigorously focused on instrumental and normative ends (the use of clinical research to improve outcomes), and their emergence coincided with emerging foci on accountability and cost containment, and more recently foci on value for money and accountability for performance in health services. Although not yet apparent in this decade, it is likely that subsequent decades if mapped with a wide enough net will reveal an explosion of related fields within EBM, comprised at least of quality improvement and safety subsets. EBM adherents may represent a new epistemic culture of knowledge production [144].

We propose that it is the EBM Group's emphasis on systematic reviews and their active dissemination and transformation, coupled with a highly normative and prescriptive orientation, that creates the conditions for this new form of knowledge production. Rogers, working without the aid of computer databases (for much of his early career at least) also synthesized, pouring laboriously through all known diffusion studies. In this regard there are similarities. Rogers' goal, however, was a synthesis and representation of a general theory of innovation diffusion. EBM's goal is prescriptive while being rigorously empirical – to guide and inform medical practice, working from a model in which the 'gold standard' in medical knowledge has been defined. Rogers sought to understand the process of how new innovations were diffused. EBM may be creating by example a new way of diffusing innovations. Its members have used the innovation diffusion literature and are linked in many ways to this literature. But this epidemiology based group has not as yet evidenced an intent (nor we argue should they necessarily) to build theory about knowledge utilization, technology transfer, or innovation diffusion. Rather, it is highly prescriptive and characterized by a strong underlying assumption that practitioners of EBM have (or can get) the best knowledge and the best knowledge production model. They are linked to the original problematic of innovation diffusion through the age-old problem of the know-do gap [149].

## Limitations

Most of the limitations of this study are typical of bibliometric studies generally, including the inclusiveness of the Web of Science, inaccuracies in the databases, analysis by first authors only, and limited knowledge of the context of citation. We chose the Web of Science because it contains all of the necessary fields to conduct bibliometric analysis and is a multidisciplinary database [150]. The Web of Science, however, does not represent all disciplines equally and therefore knowledge utilization articles within sciences or health care are more likely to be represented than knowledge utilization articles within the social sciences. Publication counts, such as number of publications by author or country, number of journals, etc., are therefore biased toward science and medicine. However, citation analysis such as ACA is not affected by the journals indexed in the Web of Science, because all cited documents are listed regardless of whether or not the journal is indexed in the database. Spelling and bibliographic variances are common in databases [151], and can cause errors in publication counts. We corrected for these variances by undertaking a detailed manual review and correction of variances. Lastly, conducting analyses by first authors attributes all contributions of a work to only the first author.

Three specific criticisms are leveled at author co-citation. First, resulting maps may omit authors an informed reader may view as central [50]. In this case, the reader may disagree (as he or she is free to) with our judgment sample. Second, such maps may fail to reflect an informed reader's knowledge of new directions to which authors' recent papers and interests may have led [50]. This is unavoidable, but such directions can be traced and assessed with future analyses. Third, a more fundamental epistemological criticism exists – one which asks whether the maps yield the true picture. However, it is unlikely that there is any one true picture; all maps leave some information out, and include other information. If a reader's views differ from ours, whose view is preferred? In response, White has argued: "... the status of the maps is neither preferred nor nonpreferred *a priori*; it must be decided in light of the claims being made and the overall evidence brought to bear" [[50], pp. 100–101]. It is also the case however that these maps have been quantitatively validated as being quite congruent with data collected independently from scholars in the field [52]. Finally, given publication delays of sometimes up to two years, it is unlikely that we have captured the state of science in the field beyond about 2002. A limitation of this and similar studies is the effect of partitioning on papers/author/journals published toward the end of the decade. These have a lower likelihood of being cited than do those published toward the beginning of the decade. We could also have restricted the cited year window, by only accepting citations, for example, not older than 10 years. Had we done this, it would have resulted in a more dynamic picture focusing more attention on currently active scholars. However, we were interested in an historical, longitudinal mapping, and including older citations was important to see work that has continued to be cited actively.

## Conclusion

In this paper, we used ACA to show that new forms of knowledge production and utilization – in particular, EBM – are emerging and are rapidly accelerating. It might seem that EBM has no relation to the hybrid corn study of so long ago, or innovation diffusion as a field. Our longitudinal analysis shows clearly, however, that this new field of activity in the medical sciences has strong intellectual roots and is indebted to the social science discipline of innovation diffusion. The relevance of such a finding lies in the particular pattern of emergence of EBM. We argue this pattern is representative of a broader societal shift to a different form of knowledge production than has characterized innovation diffusion for almost six decades. With this shift, the social contract between science and society is undergoing a renegotiation, a renegotiation with society as a far more active partner in the creation of knowledge.

Gibbons and Nowotny [1,152] have characterized this renegotiated form of science as mode two knowledge production. They have described it as involving non-hierarchical relationships with stakeholders, such as industry, government, and health care decision-makers. Its features are: knowledge production in the context of application, transdisciplinarity, a much greater diversity of sites of knowledge production, high reflexivity, and novel forms of quality control [123]. Such mode two knowledge production, based on the needs of end users in the health care system, is arguably a more socially accountable form of knowledge production. This is in contrast to mode one production that reflects what have historically been the traditional, academic norms of scholarship in the disciplines and institutions in which researchers work, such as academic tenure and promotion based on high impact, peer-reviewed publication [1]. The foundations of mode one production rest on principles of scientific expertise, peer review, and non-interference.

The health care environment is characterized by ever increasing demands for accountability in the wake of troubling reports suggesting that quality of care is less than optimal [12,14]. This, coupled with unusually high volumes (relative to the social sciences, for example) of peer reviewed outputs from elite medical researchers, creates at least two of the necessary conditions for the rapid emergence of a new domain, namely EBM. The study of this new domain will be of interest to a wide range of scholars, for example those interested in bibliometric methods, those studying the sociology of knowledge, and those engaged in science studies. The emergence of EBM is also a potentially compelling story in its own right, and one deserving of a detailed examination. The first 15 years of its history currently reside in its artifacts (a central example of these artifacts being peer-reviewed papers). Its originators are also still actively engaged in creating its history. In this regard, we can do better than history of the cliometric sort suggested by White and McCain [[49], p. 327]:

"Because the data of ACA are merely noun phrases and associated citation counts, they produce history of the cliometric sort, which leaves out almost all the good parts, such as who had shouting matches, who slept with whom, and what actually gave rise to the most significant work."

Few social science studies provide the "good parts", or the good parts are written out in an attempt to make a qualitative analysis sound more objective. In this analysis we argue that the emergence of EBM is a striking example of a shift in knowledge production mode that is actually a "part" worthy of closer examination.

## Competing interests

The authors declare that they have no competing interests.

## Authors' contributions

CE conceived the study and its design, secured funding, provided leadership and coordination, participated in data analysis and interpretation, drafted the final manuscript, and approved the final submitted manuscript. LD conducted data analysis and made major contributions to interpretation of findings, writing, and in providing critical commentary. JL participated in designing the study, securing grant funding, participated in all team meetings providing critical and substantive commentary to both process and final products. CW coordinated and conducted all searches, ran all analyses, produced all maps, figures, and tables, and contributed to writing the final manuscript. LW, SS, and JPM participated in study design, all team meetings and data interpretation; provided input into the writing of the manuscript. All authors read and approved the final manuscript.

## Supplementary Material

Additional file 1 **Search strategy.** A complete search strategy used in the Web of Science to obtain bibliographic data for this bibliometric study Click here for file

Additional file 2 **Detailed methods.** A more detailed description of the bibliographic/ACA methods used in this study Click here for file

Additional file 3 **Detailed findings.** A more detailed description of the bibliographic and longitudinal ACA findings of this bibliometric study Click here for file
