# Supplementary material for: The intellectual structure and substance of the knowledge utilization field: A longitudinal author co-citation analysis, 1945 to 2004
Source: Implement Sci. 2008 Nov 13;3:49. doi: 10.1186/1748-5908-3-49 (PMC2621243; doi:10.1186/1748-5908-3-49)
Supplement: Additional file 1 — Search strategy. A complete search strategy used in the Web of Science to obtain bibliographic data for this bibliometric study [file 1748-5908-3-49-S1.doc]

# Search strategy

**knowledge** use OR knowledge utili*ation OR knowledge uptake OR knowledge transfer OR knowledge mobili*ation OR (knowledge AND (disseminat* OR exchange OR implement OR translat* OR adopt)).ti OR knowledge diffusion

**OR research** utili*ation OR (research AND (uptake OR use)).ti

OR research transfer OR research translat* OR research disseminat* OR research implement*

**OR evidence** based medicine OR evidence based practice OR

(evidence based).ti OR evidence uptake OR evidence disseminat* OR (evidence AND implement*).ti

**OR diffusion of innovation**

**OR guideline** implement* OR guideline use OR guideline utili*ation OR guideline uptake OR guideline exchange OR guideline translat* OR guideline disseminat* OR guideline adopt* OR guideline mobili*ation OR (guideline AND (transfer OR diffusion).ti

**OR (idea*** AND (diffusion OR disseminat*OR mobili*ation OR transfer OR exchange OR translat*).ti

**OR Science** utili*ation OR (science AND (adopt* OR diffusion OR disseminat* OR mobili*ation OR transfer* OR translat*).ti

**OR Health technology assessment** AND (implement OR polic*)

**OR Systematic review** and (use OR utili*ation OR uptake OR implement* OR transfer* OR mobili*ation OR exchange OR translat* OR disseminat* OR adopt* OR diffusion)

**OR (Innovat*** use.)ti OR innovat* implement* OR innovat* exchange OR innovat* translat*OR innovat* mobili*ation OR innovat* adopt* OR innovat* diffusion OR

(innovat* AND (transfer OR uptake OR utili*ation OR disseminat*).ti

**OR Technolog*** mobili*ation OR **technology transfer** OR technolog* adopt* OR technolog* AND (uptake OR implement* OR disseminat*).ti

**OR information** utili*ation OR information implementat* OR information uptake OR information adopt* OR information mobili*ation OR information translat*

OR (information AND (disseminat* OR exchange OR transfer OR use).ti

**OR theor*** use OR theor* utili*ation OR theor* uptake OR theor* implement* OR theor* transfer **OR** OR theor* mobili*ation OR **theor*** adopt* OR theor* diffusion OR

theor* AND (disseminat*).ti

Simple Boolean searching of all the terms of interest (knowledge, research, evidence, guideline, ideas, science, innovation, technology, information, theory AND use, utilization, uptake, implementation, transfer, mobilization, exchange, translation, dissemination, adopt, diffusion; technology transfer; diffusion of innovation; health technology assessment; systematic review; evidence based practice) resulted in over 100,000 hits (WoS has a limit of 100,000 hits). In order to begin to limit the number of hits and increase precision of the search, the search strategy was developed and tested by scanning each combination of the terms & phrases of interest for relevance. Some terms were combined using AND, some were used as phrases and some were limited to title. Some combination of terms were not included in the search strategy as the results were not relevant to our study. For example, the phrase Research adopt* was excluded as this search resulted in citations about research into adoption, and Evidence transfer was also excluded as the citations related to forensics.
