# Supplementary material for: The intellectual structure and substance of the knowledge utilization field: A longitudinal author co-citation analysis, 1945 to 2004
Source: Implement Sci. 2008 Nov 13;3:49. doi: 10.1186/1748-5908-3-49 (PMC2621243; doi:10.1186/1748-5908-3-49)
Supplement: Additional file 2 — Detailed methods. A more detailed description of the bibliographic/ACA methods used in this study [file 1748-5908-3-49-S2.doc]

# The intellectual structure and substance of the knowledge utilization field: A longitudinal author co-citation analysis, 1945 to 2004

**Detailed methods**

This file contains detail on the methods used that could not be included in the manuscript.

**Methods background**

**Intellectual mapping using citation analyses**

Bibliometric analysis (bibliometrics) provides insight into the growth of literature and the flow of knowledge within specific fields of scientific inquiry. It uses citation data and quantitative analysis to trace published literature and to study the patterns of publication within a field. Hulme described it as “statistical bibliography” [1]; Merton and Garfield described it as “scientometrics” [2]. Pritchard introduced the term bibliometrics in 1963 and described it as “the application of mathematical and statistical methods to books and other means of communication” [3, p. 349]. It has been used to analyze scientific products, offering an objective, quantitative evaluation of rapidly growing bodies of literature [4]. It has been used to evaluate the products of science and technology [4] and to outline the history and structure of a field, including patterns of collaboration among scientists [5]. Moed outlines five general purposes to which bibliometric methods are applied [6]: Assessments of the contributions made by various individuals, groups, and institutions to advancing knowledge; analyses of global scholarly systems; analysis of scholarly fields; analyses of the science-technology interface and economic contributions of science; and analyses of education, social, and cultural contributions of basic research.

His third purpose is the focus of this paper. In analyzing scholarly fields, investigators map structures over time using techniques such as co-citation, co-word, and author co-citation analyses [6, Chap 1]. Recently, longitudinal analyses of the structure and evolution of fields have been reported by White and McCain [7, 8]. Several reports have argued that co-citation maps/citation analyses are powerful tools for mapping the intellectual structure of a field over time (8-11). In our work, we used author co-citation analysis (ACA) in the manner of White and McCain [8] to map changes in the intellectual structure of the knowledge utilization field over time.

**What do citations measure?**

Moed argues that there are at least five disciplinary viewpoints from which citations are studied and their meanings constructed: physical, sociological, psychological, historical, and information/communication**-**scientific [6]; he argues further that scholars often inhabit more than one school of thought. In this paper our use of co-citation analysis is most closely aligned with information scientists Small [9],White [10], and McCain [11]. The understanding that co-citation analysis can be used to represent the intellectual structure of a field originates with White and McCain [8]. From Small [12] we adopt the idea that highly cited documents become “concept symbols” in their fields. We have also been influenced in our interpretations by different sociological perspectives, among them sociologists Harriet Zuckerman [13] who viewed citations as markers of intellectual influence, and Robert Merton who viewed the act of citing as both reward and payment of intellectual debts [14], as well as constructivists Bruno Latour [15] and Michel Callon [16] who view citation as a way of “enrolling allies” to strengthen one’s own knowledge claims.

Merton [14] argued that science was a special institution whose members adhered to a special set of norms — communism, universalism, disinterestedness and organized skepticism [17, 18]. In his view, scientists’ only reward for knowledge production was intellectual property rights, given by the acknowledgement of priority of discovery [18]. Scientists claim their property rights by offering their knowledge freely, and they receive the reward of recognition in part through citation of their work by others [19]. The individual scientific practice of citing the work of others is controlled strongly by institutional norms going back three centuries “to acknowledge on whose shoulders they stand” [20, p. 439]. Citation is a positive sanction which helps individuals to avoid the negative sanctions of plagiarism, and functions to keep scientific knowledge flowing freely.

Latour views stable knowledge as the outcome of strong actor networks formed partly by “enrolling allies” [15]. By citing another’s work, an author strengthens his or her own knowledge claim by tying it to those cited, and anyone wishing to contest the knowledge claim must also take on all the stabilized knowledge claims represented by the author’s citation list. The social process of making knowledge consists of the successful alignment of initially diverse claims, and if the network is strong enough, the author’s knowledge claim becomes an obligatory passage point [16]. Any future authors wishing to make claims on the topic must “go through” this passage point (the author’s work), which is done most frequently by citation. In attempting to empirically test the constructivist versus normative explanations, Baldi argued that his evidence supported a normative interpretation of citations [21]. Our perspective however, is more congruent with that of Small, who argued that both normative and constructivist interpretations of citation patterns are valid [22].

**Author co-citation analysis**

In ACA, frequently cited and co-cited authors are the unit of analysis, and the actual maps in ACA represent an author’s citation image [8]. White and McCain argue that:

“The decisive argument for ACA is that it enables one to see a literature-based counterpart of one’s own overview of a discipline. In our experience, the agreement between the computed and the private overview is generally quite good. We thus have an answer for the person who looks at our graphics and says “I know all that already.” If that indeed is the case, then we have made technical progress, since we can now reproduce much of the disciplinary expert’s view on behalf of someone who does not know as much, and we can do it without benefit of the expert” [8, p. 329].

White and Griffith [23] are credited as having published the first ACA showing intellectual structure of the information science field. White and McCain [8] used ACA to look longitudinally at the field of information science (1972 to 1995). They argued that change over time was not a given, reporting “overall stability of information science” across the period of study. They concluded that in their case ACA was useful for “rendering the inertia of fields” [8, p. 342], noting that their longitudinal maps did not differ from the original map of the information science literature produced by White and Griffith [23]. The co-citation patterns of a global group of scholars with varying degrees of social ties shows that citation patterns do not follow friendship or acquaintance ties:

“Not only is there an independent effect of intellectual affinity, when it is present there is no effect of social or collegial ties. Although co-citation may well have led to social or collegial relationships in the past — as co-cited authors notice each other and develop contact through conferences and correspondence — it is the intellectual affinity reflected in co-citation that the regressions point to, and not the social ties. As a direct effect, it is intellectual affinity, what they know, that matters and not social ties, who they know” [24, p. 124, original emphasis ].

Baldi also found evidence that authors are cited for the content of their work, rather than citation reflecting relative prominence within a field [21]. The implication is that prominence is the outcome of intellectually important work.

Cottrill, Rogers, and Mills reported an ACA examining sets of works from the diffusion of innovation and the technology transfer literatures (1966 to 1972), using sets of documents (oeuvres) of 90 authors drawn from the diffusion literature, and 75 from the technology transfer literature [25]. They found overlap between the works of the authors, *i.e.*, people belonged to more than one specialty. Using multi-dimensional scaling and content analysis, they clustered the 110 authors from the two major fields into five subfields: technological innovations and economic growth, organizational innovativeness, diffusion research, communication of scientific and technological information, and geography and quantitative methods.

**Invisible colleges**

Another use of co-citation analysis is the identification of invisible colleges [5, 26] — groups of elite, interacting, scientists who may be geographically dispersed but who exchange information to monitor progress in their field [27-29]. These invisible colleges unify and provide coherence to a research field [30] by mediating large proportions of the informal communication within a field with those most central scholars having most access to critical communication channels [31]. Although the concept dates back to the 1600s in England with the origins of the Royal Society, and the literature is replete with definitions of invisible colleges [29, 30], they are generally agreed to represent social networks or significant thought (*i.e.*, cognitive) collectives within a field. The former are commonly studied with sociometric methods, the latter with bibliometric methods. Invisible colleges are aligned with the major domains in a field. Hence, a college of diffusion scholars is aligned with the diffusion of innovation domain and so forth. The emergence or strengthening of an invisible college on one hand or the weakening or loss of one altogether on the other, signal important changes scientifically and intellectually — potentially serving as indicators that may herald significant changes in the ongoing negotiations between science and society of their (sometimes uneasy) social contract. Although authors who are frequently co-cited may communicate or even know each other, author co-citation as a method maps intellectual structure and does not provide direct evidence of social networks in a field.

**Methods**

**Search strategy**

We searched the Web of Science online database covering 1945 to October 2004 utilizing combinations of keywords derived from concepts within the scope of the study and downloaded bibliographic information from 14,968 papers. The number of documents for 1995-2004 was annualized in the publication counts, with October 27 being day 300 of 365 days. The goal of the search was for a balance between recall (exhaustivity) and precision (specificity). Recall is the number of relevant documents retrieved compared to the total relevant documents [32]. To ensure high recall, we took additional steps beyond searching with our search strategy. We first reviewed reference lists of key works and second, we searched for the works of key authors. Reference lists were searched that represented health and were relevant to clinicians, managers, and policymakers and were chosen by the investigator group based on expert opinion. Key author names were also searched. An initial list of key players in the knowledge utilization field was compiled and then sent to current experts in the field for review. The list of experts chosen represented different countries, target audiences, and domains of knowledge utilization to ensure a representative and comprehensive list. Additional searching using the reference lists and key author names resulted in an additional 928 titles being downloaded. We calculated our recall at 88.7%, based upon how many of the possible 200 most cited documents were retrieved in our initial search.

Precision is the number of relevant documents retrieved compared to the total documents retrieved [32]. We addressed precision by reviewing all titles and screening for inclusion/ exclusion based on predetermined decision rules. These rules are included in the technical report, available on request. The titles were reviewed for inclusion/exclusion into the study by the project coordinator (CW) and a group of graduate students studying in the area of knowledge utilization who were paired with an investigator. All pairs had an inter-rater agreement of more than 80%. A total of 7,183 titles were excluded based upon review of the titles using the decision rules for inclusion and exclusion. As a final precision check, the first author reviewed all titles that were excluded from the study to ensure that no titles were inappropriately excluded.

**Data management**

Data cleaning involved removing 336 duplicates using the Bibexcel [33] freeware’s “remove duplicates” feature; we supplemented this with a manual review of the titles. A total of 3,099 titles that were not “articles” (from the document type field) were excluded, as articles most often represent new scientific production in a field of study [34, 35]. From the initial download of 14,968 titles, 5,278 articles were retained. The data files were cleaned prior to analysis by correcting for variation in author name, cited author name, cited documents, journal name, and country. The data files were corrected for variations such as the British Medical Journal being identified as both the “British Medical Journal” or “BMJ”, authors such as Everett Rogers identified as E. Rogers or E.M. Rogers, USA was not identified, but rather states were identified. The data were categorized by decade, according to the year of publication of the document, resulting in separate files for the periods 1965 to 1974, 1975 to 1984, 1985 to 1994, and 1995 to 2004.

Prior to analysis, each title was also reviewed by the project coordinator (CW), a health sciences librarian with experience in the knowledge utilization field, and classified according to one or more domains of knowledge utilization. The ten domains of knowledge utilization were created by the investigators based upon review of the most frequent keywords used in all of the papers. The domains included knowledge utilization, policy, evidence-based medicine, technology transfer, diffusion of innovation, guideline, systematic review, knowledge management, decision-making and other. The domains were not mutually exclusive; each title could appear in more than one domain.

**Analysis**

Maps were produced for each decade using the twenty-five most cited authors. The goodness of fit between the map and the data matrix was measured using Kruskal’s Stress 1 measure [8] as a reflection of goodness-of-fit, with a stress value of 0.2 considered to be acceptable [11]. Our values for Kruskal’s Stress 1 measure were 0.06, 0.16, 0.12 and 0.13 for each of the decades respectively. The two-dimensional author co-citation maps included circles or nodes that represent frequency of author citations, and lines joining the circles represent author co-citation [11, 36]. Thicker lines and closer nodes indicate that the pair are co-cited more frequently, and therefore their work is considered to be conceptually similar [36]. These visual maps represent the “important structural features, themes, and relationships in the literatures being studied” [37, p. 125].

To interpret the maps, lists of the cited documents for each of the most cited authors, information about the domain of study, and biographical information about the author were compiled. Using this information, and expert knowledge of the field, the domains of knowledge utilization were overlaid onto the co-citation maps for ease of interpretation of the maps. We present co-citation maps by decade (1965 to 2004); these aggregate the citation behavior of individual citers, resulting in a visual representation of the intellectual structure of the field [23]. The first map is for the decade of 1965 to 1974 because prior to that there were insufficient authors to create meaningful maps, but our analysis starts in 1945.

**Descriptive findings — core journals**

Bradford’s Law of Dispersion or Scattering [38] asserts that the core journals in a field can be divided into three groups, with each group containing the same number of articles. In a given field, the first group will comprise a relatively small number of prolific, or core, journals. The second group comprises a larger group of journals, and the last group includes a much larger number of journals [38].

The 5,278 articles in the analysis were published in 1,897 journals. The journals were rank ordered according to the number of articles they had published from the dataset. Consistent with Bradford’s law, there were 95 “core” journals, representing the location of most published research on a topic, 407 journals in the middle zone, and 1,395 journals in the lowest zone.

**References**
