# Supplementary material for: The intellectual structure and substance of the knowledge utilization field: A longitudinal author co-citation analysis, 1945 to 2004
Source: Implement Sci. 2008 Nov 13;3:49. doi: 10.1186/1748-5908-3-49 (PMC2621243; doi:10.1186/1748-5908-3-49)
Supplement: Additional file 3 — Detailed findings. A more detailed description of the bibliographic and longitudinal ACA findings of this bibliometric study [file 1748-5908-3-49-S3.doc]

# The intellectual structure and substance of the knowledge utilization field: A longitudinal author co-citation analysis, 1945 to 2004

**Longitudinal findings in greater detail**

This file contains a more detailed version of the longitudinal findings than could be included in the manuscript.

**The field over time**

The evolution of the knowledge utilization field by decade illustrates the emergence of new and more robust domains over time. Collins has argued that relatively small numbers of privileged scientists exist at the “coal face” of knowledge production. These scientists produce their “scientifically certified knowledge” through not only experimentation but also the resolution of scientific controversies. Collins labeled these scholars “core sets” [1, 2], distinguishing these core sets from collections of scientists such as “invisible colleges” who are closely connected in a variety of ways. He argued that core set members are not necessarily in frequent or sustained contact. So while Collins’ understanding of core sets differs significantly from the idea of invisible colleges in this paper, it is useful to the extent that it enables us to identify a small group of scholars who were actively engaged in the production and certification of knowledge.

The authors we identified as core set authors are represented in the maps in Figures 1 to 4 and highlighted in Table 5 by decade. The core set of scholars on the map in the first decade (1965 to 1974) are from very different disciplines (sociology, economics, geography, management, information science), but are linked by their work in innovation diffusion. The maps show that over time, the members of this core set become central figures in the distinct subfields which represent their original disciplinary orientation. However, the maps also show that no theory of innovation diffusion supplants that represented by Everett Rogers (1931 to 2004).

**1965 to 1974**

The first first-author co-citation map (Figure 1) shows a cohesive group of co-cited authors who come from disparate disciplines [3]. They are linked by their common focus on aspects of the diffusion process and the gap between research and practice, and constitute the core, or parent domain of diffusion of innovations. The largest, most central node belongs to Everett Rogers, who in this decade published two editions of his groundbreaking work “Diffusion of Innovations” [4] (the second edition was titled “Communication of Innovations: A Cross Cultural Approach” [5]). This work marks the first analysis of all known diffusion studies [4, 5], and also the first, and most successful, attempt at a general theory of diffusion. The citation patterns show that from the outset, Rogers’ representation of innovation diffusion theory constituted the main paradigm guiding intellectual work in diffusion of innovations.

Above Rogers’ node is Elihu Katz, a sociologist and diffusion researcher who was linking disparate fields of diffusion research, such as communication and agricultural innovation [6, 7]. Katz’ and Rogers’ nodes are close to and strongly linked to the nodes of sociologists James S. Coleman and Herbert Menzel, who worked with Katz on the social aspects of the diffusion among doctors of the new antibiotic tetracycline [8, 9].

Below Rogers’ node, and close to it, is Edwin Mansfield, an economist then writing about the diffusion of innovations in business firms [10-12]. Mansfield is linked to both Rogers and another economist, Zvi Griliches, who examined the economic factors affecting the diffusion of hybrid corn [13]. Directly below Mansfield is Thomas J. Allen, whose work is linked to Rogers through Mansfield. In this period, Allen studied research and development organizations, looking at how engineers and scientists communicated and solved problems in organizations [14]. Although all three of these scholars were associated with technology transfer, the content of their work was quite different from each other [15, 16].

To the right of Rogers, and strongly linked to him and to Griliches, are geographers Torsten Hägerstrand and Lawrence Brown, who researched the spatial aspects of diffusion theory [17-19]. Hägerstrand also did research in the diffusion of agricultural innovations, using Monte Carlo game theory to simulate the diffusion of farm practices [20, 21].

To the left of Rogers are sociologist Alvin Gouldner, management theorist W. Jack Duncan and philosopher C. West Churchman. Gouldner [22] studied the differences between “cosmopolitans” (people who went fairly often to a larger city) and “locals”, and the roles that they played in organizations. Duncan wrote widely in the area of organization and management theory, studying how to transfer management theory to practice [23]. Churchman studied the gap between managerial decisions and scientific knowledge [24, 25]. At the very bottom of the map, very distant and not linked to the rest of the scholars, is Gerard Salton, an information scientist who examined the link between information dissemination and automatic information systems [26, 27].

**1975 to 1984**

The map of this decade shows a rapid uptake of diffusion scholarship by authors from a variety of fields. The parent domain diffusion of innovations grows; Rogers’ node remains the largest on the map. This decade also shows the emergence of two new branches from the parent domain: technology transfer and knowledge utilization (Figure 2).

**Diffusion of innovations**

Everett Rogers’ node is again the largest and most central on the map. Sociologists James Coleman and geographer Lawrence Brown remain with Rogers in the parent domain of innovation diffusion.

**Knowledge utilization**

Although it appears as if the domain of knowledge utilization developed out of nowhere, key scholars in this field, Edward Glaser and Ronald Havelock were among the most cited authors in the previous decade (1965 to 1974) but were not on the map (they appeared when we permitted 50 authors). The conceptual center of this new domain is the work of a new group of scholars: Carol Weiss, Nathan Caplan, and Robert Rich. In this decade they share a research focus on the use of social science research in public policy [28-30]. They are also all strongly linked to the parent domain of diffusion of innovations, particularly to the work of Everett Rogers. The large number of links between clusters within this domain indicates that there is a high degree of use of these scholars’ ideas by citing authors. The field is also fairly tightly clustered, suggesting a high degree of conceptual similarity among authors.

Moving out from the center, we find the nodes of Edward Glaser, Ronald Havelock, and Robert Yin. Havelock’s earlier research [31-33] examined how knowledge could be used to plan for innovation. Almost 15 years later, Glaser followed on this theme by co-authoring the influential “Putting Knowledge to Use: Facilitating the diffusion of knowledge and the implementation of planned change” [34]. Yin’s research is conceptually different, focusing on how new practices become routine [35], and the role of networking in knowledge utilization [36].

On the other side of the central core are Mark van de Vall, Ian Mitroff, and Robert Merton. Van de Vall’s main research interest in this decade was on the theory and methods used in applying social science research [37, 38]. Merton, although known for his work in the sociology of science, particularly the “Matthew Effect” [39] is cited in this decade for the first and revised editions of his book “Social Theory and Social Structure” [40, 41], and for his work on focused interviewing [42]. The links on the map show that he is fairly strongly linked to fellow sociologist James Coleman, in part at least because their careers overlapped — Merton was at Columbia from 1941 to 1984 and Coleman received his PhD from Columbia in 1955. Merton had done previous research in medical sociology on the training of student physicians [43]. Ian Mitroff was also a sociologist of science. In this decade he was most cited for his 1974 book “The Subjective Side of Science” [44], where he examines the wide gap between the finished products of scientific work (publications) and the actual processes of forming knowledge. Mitroff is particularly important for identifying “counter-norms” (counter to Merton’s set of norms) which also guide scientific practice. For example, the norm of disinterestedness exists alongside the counter-norm of being deeply, personally committed to one’s own work and theories.

**Technology transfer**

The domain of technology transfer is characterized by a relative lack of links between individuals within the domain, and more links back to the domain of diffusion of innovations. There is no one single conceptual core in this field in this decade. Mansfield and Allen have moved from the parent domain of diffusion of innovation. However, as noted above, and by the citation lines, their work is quite different, as each is more closely linked to the domain of diffusion of innovations than they are to each other.

The economist Mansfield has the largest node, his work is at the “geographical” center of the field and has the most links to others’ works. However, even with Mansfield whose work differs significantly from that of Rogers, most links are back to the parent domain of diffusion of innovations. Mansfield’s top citations are to works from the late 1960s and early 1970s that examine the economic aspects of technological change in organizations [45-47].

Allen’s most cited research is focused specifically on Research and Development laboratories, including his 1977 book titled “Managing the Flow of Technology: Technology Transfer and the Dissemination of Technological Information within the R&D Organization” [14, 48, 49].

Quite distant from Mansfield (indicating conceptual distance), the work of geographer Brown is also still strongly linked to that of Rogers in the parent domain. However, Brown is linked to the economist Mansfield through the work of Mahajan. A major contribution of Mahajan and Peterson and Mansfield was to show how to fit mathematical models to diffusion data. Much of the citation to the Coleman, Katz, and Menzel [8] study can be traced to Burt's efforts to uncover these data and make them publicly available. Additionally, the Coleman, Katz, and Menzel study (on tetracycline use) is picked up by the evidence-based medicine (EBM) group in later decades explaining its re-emergence in later decades. The domain of technology transfer is quite different from both the parent domain and knowledge utilization. It also has writers that are quite conceptually distant, and whose work is quite distinct, such as the Federal Insurance Corporation, and Vernon who studied international technology transfer including the role of timing of the innovation [50]. There are also writers whose work spans domains, such as Utterback, who writes on technological innovations within organizations [51], and Aiken who writes on the effect of organizational structure on technical and administrative innovation [52].

**1985 to 1994**

Figure 3 shows three trends in the 1985 and 1994 decade. First is the emergence (at the left of the map) of EBM — see the work of Lomas, Eddy, and Haynes. Second, the domain of diffusion of innovations shrinks although Rogers’ node continues to dominate the map. While the field of innovation diffusion continues to be vibrant, most of its activity was in the style of Everett Rogers. Additionally, Rogers came out with another edition of his book in this decade. Rogers continued to be active in this decade, and in fact until his death in 2004. Several other important authors in this field in this decade have also continued to be active, among them Kimberley [53], Allen [54], and Zaltman [55]. Third, the knowledge utilization field shows signs of strengthening. The two new journals started during the previous decade created arenas in which scholars in knowledge utilization and diffusion could exchange ideas and develop the interdisciplinary application of science knowledge [15]. The emergence of these and other journals and societies as noted earlier were indicators of growing disciplinary cohesion. The domain of knowledge utilization became more homogenous than in the previous decade. Authors who remain highly cited in the knowledge utilization domain comprise the current intellectual core set of the field. Some authors whose work has not continued to be central to the domain of knowledge utilization no longer appear, among them Van de Vall, Mitroff, Merton, and Yin.

**1995 to 2004**

The map for 1995 to 2004 (Figure 4) shows a continuation of the trends that emerged in the previous decade. Most notably, the field of EBM exhibits significant growth, with the entrance of several new authors. While it appears that the other domains have gotten smaller in this decade, the maps are limited to the 25 top-cited authors. To check to see if the shrinkage was real or an artifact of the number of authors on the map, we re-ran the map with the 50 top-cited authors (available on request). That map showed even more growth in the field of EBM, but also showed the technology transfer and knowledge utilization domains continuing as vibrant.

The innovation diffusion domain appears to have retracted, although Rogers’ citation node continues to grow. On the map of the 50 top-cited authors, the only author to enter the innovation diffusion domain is Bozeman, who had previously been in the knowledge utilization domain. Other than this, even with 50 authors, the innovation diffusion domain had largely been conflated to represent Rogers’ work. Given the dominance of Rogers’ node in all decades, we argue below that Rogers is a canonical author [56], that his book “Diffusion of Innovations” is a canonical text for all the domains, and that his approach to innovation diffusion by this decade (and probably earlier) represents the dominant paradigm for conducting diffusion research. The fifth edition of Rogers’ most cited book was published in 2003; Rogers died late in 2004.The presence of such a dominant author stands in contrast to, for example, the information sciences field characterized by the absence of a strong central author [56].

In this decade, the most cited article is the index EBM paper. For the first time, this new group is named (EBM Working Group), and the term enters the lexicon [57]. The paper was published in a highly visible and accessed medical journal and included 29 members, among them, members on the map for this decade, Guyatt, Haynes, Oxman, and Sackett (chair of the group). After the article was published, the authors wrote more articles, cited the original article [58-61], toured and gave numerous talks — and asked physicians, “why wouldn’t you want to use the best available evidence to guide your practice?” Their work also coincided with emerging concerns about rising health costs and increasing accountability pressures (the timing was right), such as have been described by Nowotny and others [62-65].

Sociologist James Coleman reappears in this decade, in the domain of diffusion of innovations. He was a prominent American sociologist who focused on the sociology of education and public policy, but his early work was in the sociology of medicine. The work most cited is the well known tetracycline study in “Medical Innovation: A diffusion study” [9]. While this reappearance of older work has been described by White and McCain and may be a sign of the revival of a domain [56], we argue there is not a “revival” of a domain, but rather a reappearance of this one work, which is highly relevant to the new project of EBM. Coleman is highly cited in works related to the diffusion of innovations within healthcare [66-68].

During the last two decades, the significant majority of new people entering are in the field of EBM. Key figures exiting are Bozeman, Dunn, Glaser, and Rich from knowledge utilization; Kimberley and March move from diffusion of innovation. Glaser’s citations, for example, are all to his 1983 book that at the time was a momentous achievement in a relatively sparsely populated field. By the mid-nineties with no updated edition, not only is it cited less often, but also there are many additional papers in a rapidly expanding field and set of subfields. Over time, the separate domains show increasing conceptual cohesiveness — citation nodes move closer to each other within the field, and the fields as wholes are more easily separable from the other fields (the contrast is particularly vivid when compared to the 1965 to 1974 decade in Figure 1).

**Canonical authors and canonical works**

We identified a number of “canonical authors” whose work has enduring importance to the field. White and McCain define a canonical author as someone who appeared on the citation maps in three or more decades [56]. We define a canonical author as someone who was on at least the last three maps (1975 to 2004) and any maps prior to that. Our canonical authors were: Everett Rogers and Gerald Zaltman (innovation diffusion), Carol Weiss and Ronald Havelock (knowledge utilization), and Edwin Mansfield and Thomas Allen (technology transfer). Rogers, management scientist Allen, and economist Mansfield were the only authors who were top**-**cited in all decades, excluding 1945 to 1954. The most cited works of these authors constitute, we argue, the canonical literature of the science of knowledge utilization as it entered the 21st century. Given publication delays of sometimes up to two years it is unlikely that we have captured the state of science in the field beyond about 2002. A limitation of this and similar studies is the effect of partitioning on papers/authors/journals published toward the end of the decade. These have a lower likelihood of being cited than do those published toward the beginning of the decade. It is clear from the size of the nodes on all maps and the number of citations accrued that, over all decades, the intellectual structure of the field is dominated by the work of Everett Rogers. Rogers’ work is emblematic of the diffusion paradigm, until the emergence of EBM in the last decade. Of the four domains, only technology transfer and diffusion of innovation have authors associated with them in all four decades.

**Diffusion of innovations**

Since the 1962 publication of Roger’s now classic book “Diffusion of Innovations”, which went through five editions before his death in 2004 (1971, 1983, 1995, 2003), Rogers was and remained the central author in the broad field of knowledge utilization. Rogers’ theory has been the dominant and most consistently used theory since inception of diffusion research [69, 70]. He based each edition on an analysis of all retrievable diffusion studies, regardless of field. For some canonical authors such as Mansfield, it is only their earliest work that is top-cited in all decades. However, different versions of Rogers’ general theory are cited throughout the decades [4, 5, 71-73], suggesting that Rogers’ influence was and continues to be a fundamental force.

Gerald Zaltman’s most cited works were “Innovations and Organizations” [74] and “Strategies for Planned Change” [75]. His work although strongly pro-innovation also reflected a belief that innovation should not be accepted unquestionably. His main contribution to the field was to offer insight into the importance of individual users of innovations to the diffusion process. He proposed that to understand how innovation diffusion truly occurs we need to study demand characteristics, such as users’ ability and willingness to seek and process innovations.

Along with Katz and Menzel, Coleman conducted a widely cited diffusion study that investigated the spread of the prescribing of a new antibiotic among physicians [9]. The finding of this study highlighted the importance of interpersonal networks in the diffusion of new medications. It was a catalyst for future investigations in this area. Rogers found this work of key importance in formulating his first book on innovation diffusion. Later, Coleman advocated for a new approach to policy research, one that engaged real world problems and produced relevant action oriented solutions. He is known for his publication “Foundations of Social Theory”, and is considered one of the earliest users of the term “social capital”.

**Knowledge utilization**

Carol Weiss was most cited for her works “Knowledge Creep and Decision Accretion” [29] and “Using Social Research in Public Policy Making” [76]. She continues to publish [77, 78]. She expanded initial views of research use as an instrumental application of research to inform a decision, to include conceptual or enlightened use **—** when findings from research influence decision makers’ attitudes to and perceptions of a social problem [79]. She described several models of utilization: knowledge driven, problem solving, interactive, political, tactical, and research as part of the intellectual enterprise of society [80]. An advocate of using research findings to inform public policy, she was among the first to examine the utilization of evaluation findings in improving program processes and program outcomes [81]. Her articulation and extension of the concept “research utilization” was an important contribution to the field of knowledge utilization [80].

Ronald G. Havelock is recognized in the knowledge utilization field for his extensive work on knowledge use, change planning, and technology transfer. Author of “Planning for Innovation through the Dissemination and Utilization of Knowledge” [32], his work spanned many fields most notably education and medicine. Building on Rogers’ “Diffusion of Innovations” [4], Havelock developed a framework to aid in the understanding and improvement of the dissemination and utilization (D and U) of knowledge in the social sciences. Guided by an extensive search and analysis of the D and U literature from education and beyond, he proposed an often cited “linkage model” that connects researchers with end users in a two-way exchange of information that mutually enhances problems solving.

**Technology transfer**

Edwin Mansfield exerted a significant impact — even in later decade maps citations to his early work are high. An influential economic analyst of technology, his contributions included generation of information regarding the length of time required for firms to uptake decisions and products used by rival firms, and how this information is spread from one firm to the next. He also studied the proportion of new products and processes that are based on academic research, and the amount of time needed for such research findings to be incorporated into the commercial environment. Citations in all decades are to Mansfield’s work in the early 1960s [45-47]. In other words, he wrote some articles that became concept symbols [82] or canonical pieces.

Thomas J. Allen has made significant contributions to various fields, including technology transfer. Allen studied the ways in which formal and informal associations within organizations contribute to the diffusion of knowledge. He identified “gatekeepers” as important individuals within organizations who bring new knowledge into their organization both by reading literature and by engaging with others outside of the organization. He found that new ideas are spread most commonly through informal mechanisms, such as personal contact in small manufacturing firms. Allen also studied the influence of distance on information transfer and developed the “Allen curve” that depicts the inverse relationship that exists between distance and the frequency of communication [14, 48, 83].

Vijay Mahajan, in the department of marketing at the University of Texas at Austin, writes on developing knowledge in the areas of marketing strategies, product diffusion, and research methodology. Mahajan is responsible for adding the temporal element into a model originally designed by Blackman [84] to explain technological substitution. He has made important contributions in the area of product diffusion and has studied the diffusion process in developing countries, which he argues is an important but understudied area [85, 86].

**References**
